# Wearable Cardioverter-Defibrillator in Adult Congenital Heart Disease: A Bridge to Decision, Recovery, and Advanced Therapies

**DOI:** 10.1007/s10741-026-10638-y

**Published:** 2026-05-25

**Authors:** Berardo Sarubbi, Anna Murredda, Giovanni Domenico Ciriello, Anna Correra, Giovanni Papaccioli, Diego Colonna

**Affiliations:** 1 Adult Congenital Heart Disease and Congenital and Familial Arrhythmias Unit, Monaldi Hospital, Via Leonardo Bianchi, Naples, 80131 Italy; 2 Parthenope University of Naples, Naples, Italy

**Keywords:** Adult congenital heart disease, Sudden cardiac death, Ventricular arrhythmias, Wearable cardioverter defibrillator

## Abstract

Sudden cardiac death (SCD) remains a major cause of mortality among adults with congenital heart disease (ACHD). Risk stratification for malignant ventricular arrhythmias in this population is challenging because of the heterogeneity of anatomical substrates, lifelong haemodynamic changes, and the limited predictive accuracy of currently risk models. Furthermore, implantation of an implantable cardioverter-defibrillator (ICD) in ACHD patients is frequently complicated by anatomical constraints, limited venous access, and a significant burden of device-related complications, including infection involving prosthetic material. Several transient cardiac or systemic conditions—including haemodynamic deterioration, acquired myocardial disease, infections, inflammatory states, pregnancy, endocrine disturbances, or peri-procedural phases—may temporarily increase arrhythmic risk or impair ventricular function. In these scenarios, the indication for permanent ICD may be uncertain or premature. The wearable cardioverter-defibrillator (WCD) provides a non-invasive option for temporary protection against life-threatening ventricular arrhythmias while allowing time for clinical stabilization, diagnostic reassessment, and individualized decision-making. This review summarizes the pathophysiological rationale, available evidence, and potential clinical applications of WCD therapy in ACHD.

## Key points

Sudden cardiac death (SCD) remains a major cause of mortality in adults with congenital heart disease (ACHD).

Risk stratification for ventricular arrhythmias in ACHD is difficult because of heterogeneous anatomy and evolving haemodynamics.

Implantable cardioverter‑defibrillator (ICD) implantation may be limited by anatomical constraints and long‑term device complications.

The wearable cardioverter‑defibrillator (WCD) provides temporary protection during periods of transient or uncertain arrhythmic risk.

In ACHD, WCD therapy may serve as a bridge to recovery, improved risk stratification, advanced therapies, or definitive ICD implantation.

## Introduction

Advances in paediatric cardiology and cardiac surgery have led to a substantial improvement in survival among patients with congenital heart disease (CHD), resulting in a rapidly expanding population of adults with congenital heart disease (ACHD), who now accounts for more than two-thirds of the overall CHD cohort [1]. As this population ages, residual lesions, surgical scars, and chronic haemodynamic stress contribute to progressive structural and electrical remodelling, creating a substrate for malignant ventricular arrhythmias (VAs) and sudden cardiac death (SCD) [2].

In contemporary ACHD cohorts, SCD accounts for up to 25% of deaths, with a markedly higher risk compared with the general population [3]. Despite this burden, identifying patients who may benefit from preventive strategies remains challenging and often requires individualized assessment.

Although ICD therapy represents the cornerstone of SCD prevention in acquired heart disease, its use in ACHD is limited by anatomical complexity, uncertainty in risk stratification, and the long-term burden of device-related complications in a generally young population. Consequently, clinicians frequently encounter situations in which arrhythmic risk is clinically relevant but the indication for permanent device therapy remains uncertain.

In this context, the wearable cardioverter-defibrillator (WCD) may represent a useful option by providing temporary protection during periods of uncertainty while allowing further clinical evaluation and shared decision-making.

### Arrhythmic risk in ACHD: why decision-making is challenging

SCD is a leading cause of death in ACHD. Myocardial scar, prior surgical interventions, and residual or progressive anatomical abnormalities represent the principal arrhythmogenic substrates [4]. Over the past 20 years, major advances have been made in the management of arrhythmias in ACHD, spanning antiarrhythmic drug therapy, catheter ablation strategies, and device therapy.

Arrhythmic risk varies substantially across different CHD and is influenced by the type and timing of repair, subsequent reinterventions, and superimposed acquired cardiopathies [5]. Importantly, even patients with apparently mild disease carry a non-negligible risk of SCD. Conventional predictors derived from acquired heart disease have limited utility for SCD risk stratification in ACHD and while guidelines provide clearer recommendations for selected groups (e.g. repaired tetralogy of Fallot), they remain less definitive for many other substrates. Algorithms have been proposed for specific conditions, such as systemic right ventricle [6] and Fontan circulation [7], but have not been consistently validated in large, contemporary cohorts [8]. Moreover, discordance between risk factors and clinical outcomes is frequently observed. Changes in loading conditions, valve function, or pulmonary vascular resistance may occur over relatively short timeframes and significantly influence arrhythmic risk. Intercurrent events such as infections, atrial arrhythmias, anaemia, pregnancy, or peri-procedural phases may transiently increase vulnerability, whereas optimization of medical therapy or corrective interventions may reduce it.

Additionally, ACHD patients experience a substantial burden of ICD-related complications during both short and long-term follow-up. Reported adverse events include device and lead-related complications, thromboembolism, venous occlusion, and infections, including endocarditis. In a large meta-analysis, lead malfunction occurred in 26% of patients over 3.8 ± 0.8 years of follow-up, and inappropriate shocks were also frequent, with an estimated risk of 6.5% per year [9]. The subcutaneous ICD (s-ICD) is a safe and effective alternative to transvenous and epicardial systems [10]. However, despite avoiding transvenous lead-related complications and the risk of systemic infection, s-ICD therapy is limited by the lack of pacing capabilities and by device-specific issues such as pocket-related complications and inappropriate shocks due to oversensing [11].

As a result, clinicians frequently face challenging situations where risk is meaningful but time-dependent and poorly predictable, underscoring the need for intermediate approaches that enable ongoing reassessment and refinement of long-term management.

### Wearable cardioverter-defibrillator: general principles and evidence

The WCD is a fully extracorporeal system designed to provide continuous rhythm monitoring and automatic defibrillation for sustained ventricular tachycardia/ventricular fibrillation (VT/VF). The most widely used and validated system is LifeVest, (Zoll, Pittsburgh, PA), approved in Europe and America [12]. Other available WCD are the ASSURE system (Kestra Medical Technologies, Kirkland, WA) approved for use only in America in 2024 [13] and the Jewel device (Element Science, San Francisco, CA), not yet approved for clinical use [14] .

**Fig. 1:**
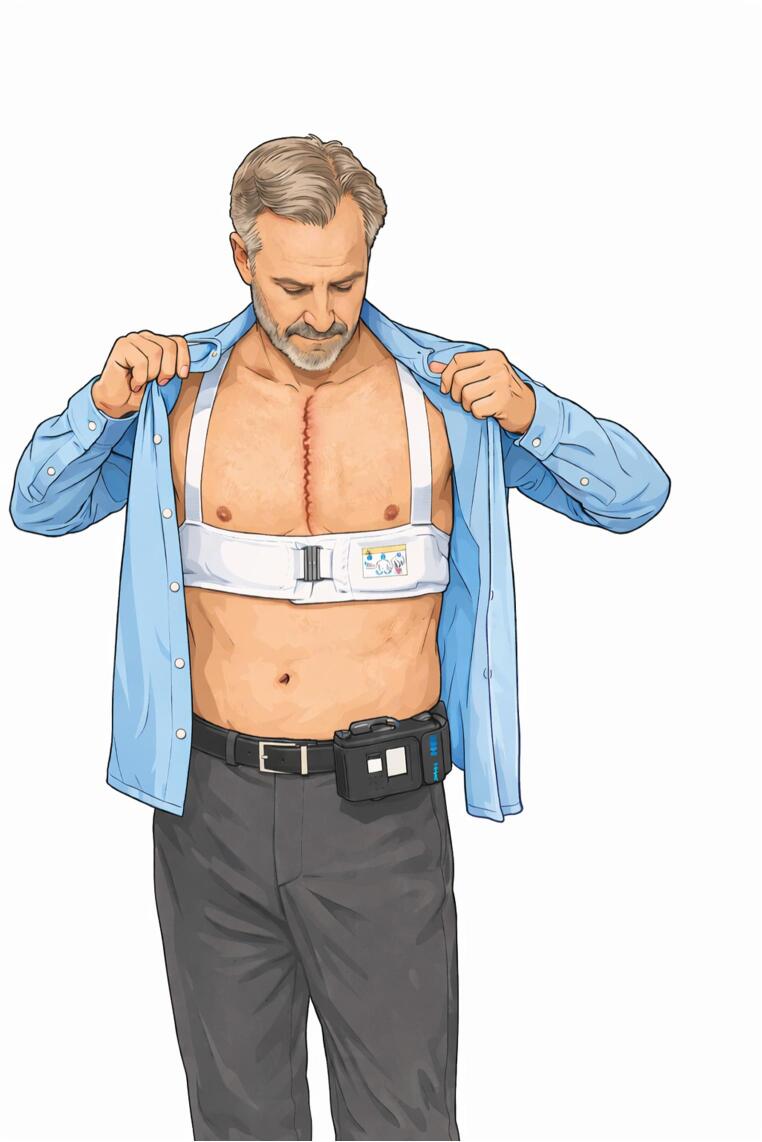
Illustrative medical drawing showing a patient with previous cardiac surgery wearing a wearable cardioverter-defibrillator (WCD)

**Fig. 2:**
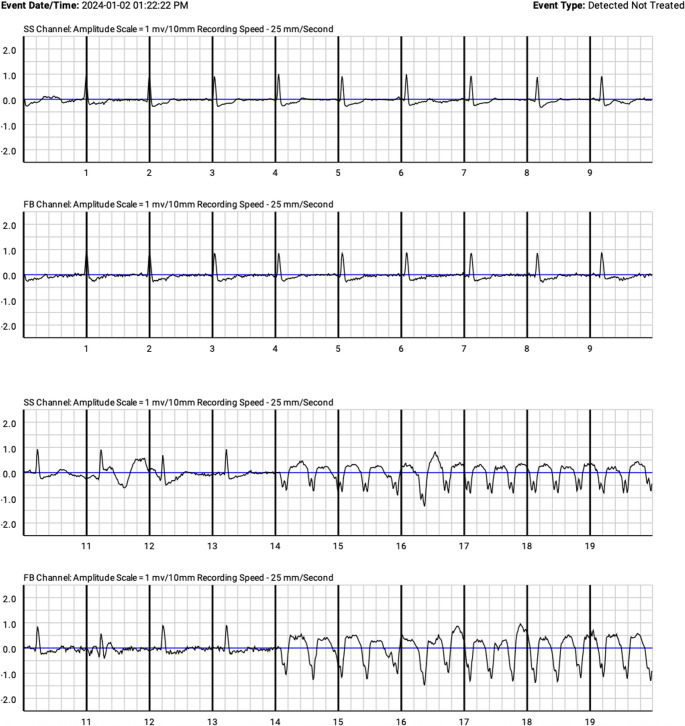
WCD rhythm recording showing ventricular arrhythmia episode

The LifeVest consists of a material vest with integrated electrodes available in five different sizes to cover all thorax diameters, and a touchscreen monitor with two response buttons, and a speaker for alerts and voice prompts (Fig. 1). The device continuously monitors the cardiac rhythm to identify sustained VAs (Fig. 2) and deliver external defibrillation when necessary.

The WCD is programmed to detect tachyarrhythmias according to predefined rate, and duration criteria, with configurable VT and VF detection zones. During detection/response time a conscious patient may abort therapy via response buttons. If the arrhythmia persists, the device can deliver up to five shocks per episode, with programmable shock energy between 75 and 150 J (default 150 J). ECG tracings, heart-rate profiles, activity data, and wear-time metrics are uploaded to a remote monitoring portal, permitting clinicians to review key information off-site and to guide ongoing management.

In current practice, the WCD is is primarily used in temporary or uncertain risk conditions, including early post-myocardial infarction phases, newly diagnosed cardiomyopathy, or situations in which ICD implantation must be deferred (e.g. after device explantation for infection) [15]. Its main advantages include immediate availability, avoidance of intravascular leads, and reversibility. Limitations include dependence on patient adherence, lack of pacing capability, and potential discomfort. Moreover, the WCD provides only defibrillation and cannot deliver bradycardia pacing, anti-tachycardia pacing, or resynchronization therapy, and logistical factors (training, fitting, follow-up and costs) further limit real-world use.

Evidence from randomized trials and large registries supports its effectiveness when worn, with a low but clinically relevant rate of appropriate therapies and high success in arrhythmia termination [12, 16–20]. However, most data derive from non-ACHD populations.

### Clinical scenarios for WCD use in ACHD (Fig. 3)

#### Temporary high arrhythmic risk: WCD as bridge to decision or bridge to recovery

ACHD patients exhibit a uniquely vulnerable arrhythmic substrate, shaped by complex cardiac anatomy and lifelong haemodynamic stress. As a result, ventricular function and arrhythmic burden may worsen in response to progression of the underlying congenital lesion or superimposed acquired cardiac disease (e.g. ischaemic heart disease or myocarditis). Such episodes may precipitate a transient decline in systemic ventricular systolic function and/or an increase in VAs, at times bringing patients into guideline-based ICD recommendations.

Newly diagnosed or worsening systemic ventricular dysfunction represents a frequent and clinically relevant scenario in ACHD population [21].

GDMT may revert ventricular dysfunction [22, 23] and have an antiarrhythmic effect in ACHD patients with a failing systemic right ventricle [24, 25]. In this subset sacubitril/valsartan demonstrated in several prospective studies to improve exercise capability, NT-proBNP levels, quality of life, and systolic function with significant increase in fractional area change (FAC) of about 3 to 6% from baseline [26–28]. Moreover, dapagliflozin further improves right ventricular systolic parameters both in symptomatic [29] and asymptomatic [28] patients with an increase from baseline to post-treatment FAC ranging from 4 to 5%.

The interplay between residual structural abnormalities (e.g. valve regurgitation or stenosis), HF and VT/SCD risk should always be considered while evaluating an ACHD patient. Following GDMT, surgical or transcatheter correction, haemodynamic may improve and ventricular size/function may partially recover, although normalization is often delayed and may require weeks to months. For example, in TOF patients with severe pulmonary regurgitation, although late valve replacement does not decrease the arrhythmic burden [30], in selected population has been associated to an improvement in RV size and a reduction in appropriate ICD therapies [31]. WCD can be offered while waiting surgical or transcatheter intervention or after the procedure for re-evaluation of arrhythmic risk. In a small case-series, WCD was used as a bridge to intervention with a good compliance and no episodes of inappropriate shocks [32].

Arrhythmias themselves can lead to HF and life-threatening VT. Atrial tachycardia, or atrial fibrillation are known precipitants of tachycardia-mediated cardiomyopathy reversible with rhythm control therapies [33]. In some ACHD with preserved ventricular function, VT related to an isthmus-dependent re-entry circuit, transcatheter ablation may potentially provide a definitive cure [34]. In all these contexts, the WCD can facilitate a structured period of reassessment—allowing optimisation of medical therapy, re-evaluation of ventricular function, and completion of additional investigations (e.g. advanced imaging, ambulatory rhythm monitoring, and, when indicated, invasive haemodynamic assessment), before committing a generally young ACHD patient to permanent device implantation and its long-term burden. This approach can also permit to postpone implantation in the most advantageous clinical condition.

Finally, arrhythmic vulnerability in ACHD may become temporarily heightened during systemic stressors. Systemic infection or inflammatory illness, endocrine disturbances (notably thyroid dysfunction), pregnancy and the peripartum period may trigger volume shifts, metabolic derangements, and autonomic imbalance. These potentially reversible perturbations can be associated with ventricular dysfunction and/or ventricular arrhythmias that may require time to stabilise. In such contexts, the WCD may be considered as a protective measure while the precipitating trigger is treated, and the patient’s clinical trajectory becomes clearer.

**Fig. 3:**
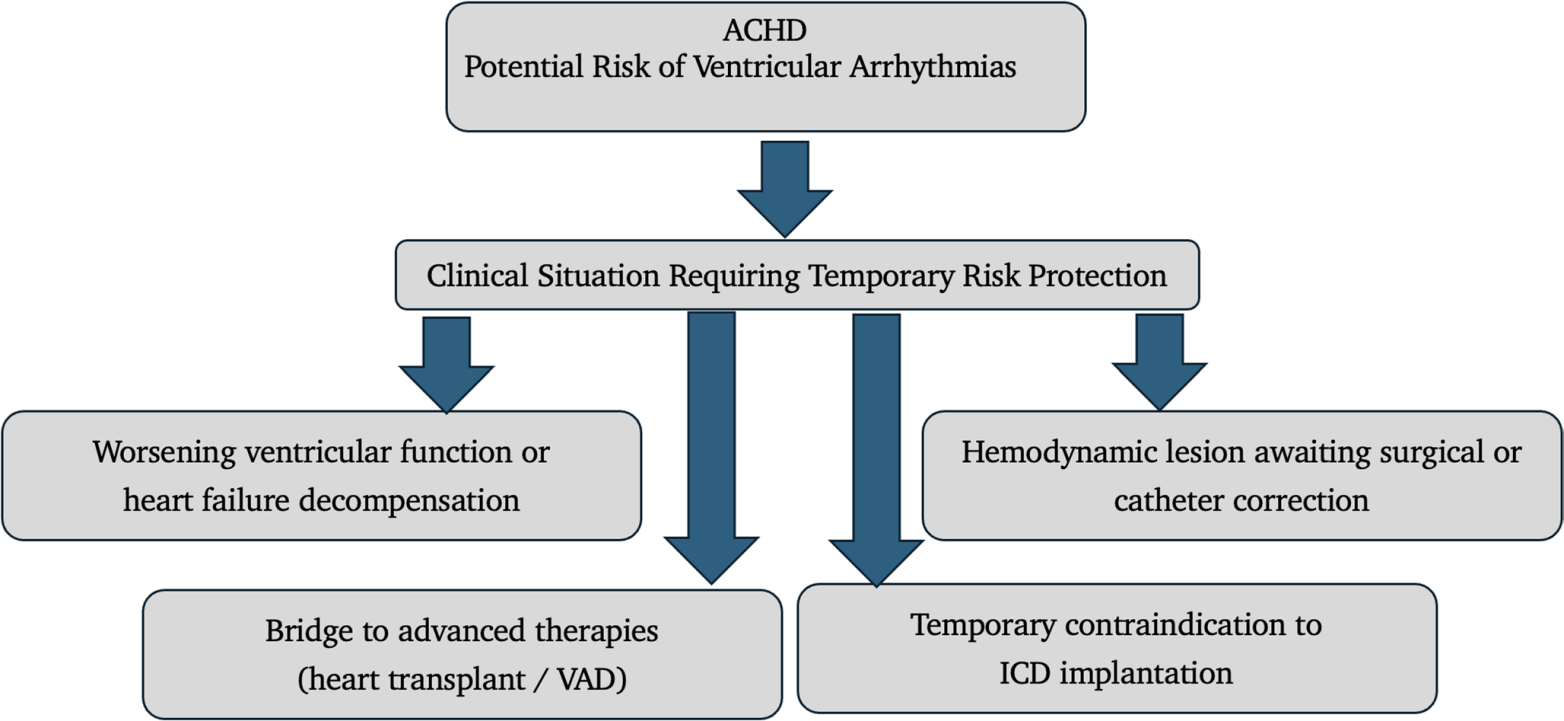
Clinical scenarios for WCD use in ACHD

#### Uncertain arrhythmic risk: WCD as bridge to better stratification

Beyond repaired tetralogy of Fallot, guidelines recommendations on ICD implantation in primary prevention of SCD in ACHD remain largely anchored to systemic ventricular systolic function, despite the limited ability of a single parameter to capture individual risk.

In everyday practice, clinicians therefore encounter patients with preserved or mildly impaired systemic ventricular function who nonetheless display features suggestive of increased arrhythmic susceptibility (such as syncope of uncertain mechanism, non-sustained ventricular arrhythmias, myocardial scar on CMR, or other high-risk markers) yet do not meet clear thresholds for permanent device implantation [35]. In these “grey zone” scenarios, a WCD may provide interim protection and monitoring while additional evaluation is completed, and risk is reclassified over time.

#### Bridge to advanced therapies (ventricular assistant device, heart transplant)

Life-threatening VAs resulting in SCD are common among patients listed for heart transplantation (HT) or ventricular assistant device (VAD) implantation.

Two wide meta-analysis demonstrated that ICD was associated with decreased mortality in patients with or without VADs in HT waitlist [36, 37]. Based on these findings International Society for Heart and Lung Transplantation guidelines for the evaluation and care of cardiac transplant candidates recommends ICD implantation in nonhospitalized patients awaiting HT in class IIa [38].

In addition, ICD implantation carries procedure-related and device-related risks, especially in ACHD population. For this reason, a temporary and non-invasive approach such as the WCD may serve as a valuable bridging strategy and is contemplated by current guidelines [19, 39, 40].This issue may be particularly relevant in ACHD patients, in whom complex anatomy and prior surgeries increase procedural complexity and infection risk.

#### Temporary contraindication to ICD: WCD as bridge to implantation

A non-negligible subset of ACHD patients have a clear indication for ICD therapy but face a temporary clinical contraindication to implantation or ongoing ICD care, most commonly infection. In this setting, a WCD represents a reasonable interim option to provide protection while the contraindication is addressed, and definitive device therapy can be resumed or reconsidered. In ACHD patients, chronic hypoxaemia and a pro-inflammatory milieu impair the host defence increasing the risk of infective processes including infective endocarditis, cardiac implantable electronic device (CIED) infection and other systemic infections [41]. In particular, the incidence of infective endocarditis among ACHD is 27–44 times that reported for contemporary adults as turbulent blood flow and prosthetic material (valve prostheses, conduits, patches, or cardiac devices) promote bacterial seeding and biofilm formation [42]. Moreover, when IE occurs in ACHD, it can be particularly severe, often involving prosthetic material, requiring prolonged antibiotic therapy, and leading to major complications such as haemodynamic deterioration, embolic events, and the need for high-risk reinterventions.

CIED-associated infection, either pocket infection or device-related endocarditis, generally mandate device removal and appropriate antimicrobial therapy, with reimplantation deferred until infection control is achieved. During this vulnerable interval, patients who remain at risk of malignant ventricular arrhythmias may benefit from a wearable cardioverter-defibrillator (WCD) as a non-invasive “bridge,” providing protection while awaiting extraction, completion of treatment, and subsequent reassessment for reimplantation.

In a large retrospective analysis of 8,058 consecutive patients undergoing ICD explantation due to CIED infection and subsequently managed with a WCD, the device showed high effectiveness in preventing sudden arrhythmic death by successfully detecting and treating episodes of ventricular tachycardia and ventricular fibrillation (VT/VF). In this registry 4% of patients experienced VT/VF events during the first 2 months and an ICD was not reimplanted in 20% of cases [43].

Less commonly, clinicians may defer ICD implantation in patients judged to be at very high infectious risk despite the absence of active infection, such as those who are immunocompromised, have ongoing or recurrent localized infections or present with significant skin/soft-tissue vulnerability at potential implant sites (including prior pocket complications or relevant cutaneous reactions). In these situations, a temporary strategy may reduce exposure to early device-related infectious complications while allowing optimization of the underlying condition.

Finally, uncertainty regarding prognosis may represent a further reason to postpone permanent ICD therapy, given that guideline-based indications generally assume an expected survival of at least one year with acceptable functional status. In selected ACHD patients with borderline or evolving life expectancy, due to advanced heart failure, multisystem comorbidity, or pending decisions regarding advanced therapies, the WCD can serve as a bridge to clinical clarification and shared decision-making before committing to a permanent device.

### Clinical evidence of WCD in ACHD patients

One of the major limitations in the literature of the WCD is the scarcity of high-quality randomized evidence. To date, only one randomized controlled trial has been conducted, enrolling patients early after myocardial infarction, namely the VEST trial [12]. Beyond this trial, the clinical evidence is largely derived from observational studies. However, these investigations are generally characterized by relatively small sample sizes and marked heterogeneity in patient populations. In addition, data specifically addressing ACHD patients remain particularly limited, as these individuals are often underrepresented and their outcomes must be extrapolated from broader, mixed cohorts rather than dedicated analyses.

In the WEARIT-II registry including 2000 patients treated with WCD, about 8.15% was an ACHD subject. In this registry the median duration of treatment was 90 days, with good compliance in all subgroups (median daily use 22.5 h). At 3 months of follow-up, patients with congenital/inherited heart disease had a higher rate of sustained VTs (3%) compared to non-ischemic aetiology. In this registry the subgroup of congenital/inherited heart disease more frequently (46% of patients) underwent to ICD implantation after the end of WCD use [19].

A prospective observational study by Rao et al. investigated the short- and long-term outcomes of WCD in 162 patients with CHD and inherited arrhythmias. In the 43 patients with CHD the predominant indication for WCD was transplant listing (35%), followed by ICD explantation for infection/malfunction (28%), ongoing cardiac evaluation (17%) and planned surgery/treatment of other medical problem (17%). No arrhythmias and no death occurred in this group while actively wearing the WCD during a median follow-up of 27 days despite a significant prevalence of left ventricular dysfunction (37%). In 32% of patients the follow-up ended for ICD implantation [44].

A case series specifically focused on ACHD patients assessed the WCD as a feasible bridging strategy. Eight patients with complex lesions were included and were monitored for up to 4 months, with a high average daily wear time (21.25 ± 1 h). No sustained VAs requiring therapy occurred, and no inappropriate shocks were delivered. Following the WCD period, half of the patients proceeded to ICD implantation [32].

Although limited, the available data suggest that WCD use in ACHD is feasible and associated with good compliance, but its true impact on hard clinical endpoints remains to be established in multicentre registry.

## Conclusions

Adults with congenital heart disease represent a complex and growing population in whom prevention of SCD remains challenging [45]. Traditional risk stratification approaches are limited by the interaction between congenital anatomy, surgical history, and evolving haemodynamics.

**Fig. 4:**
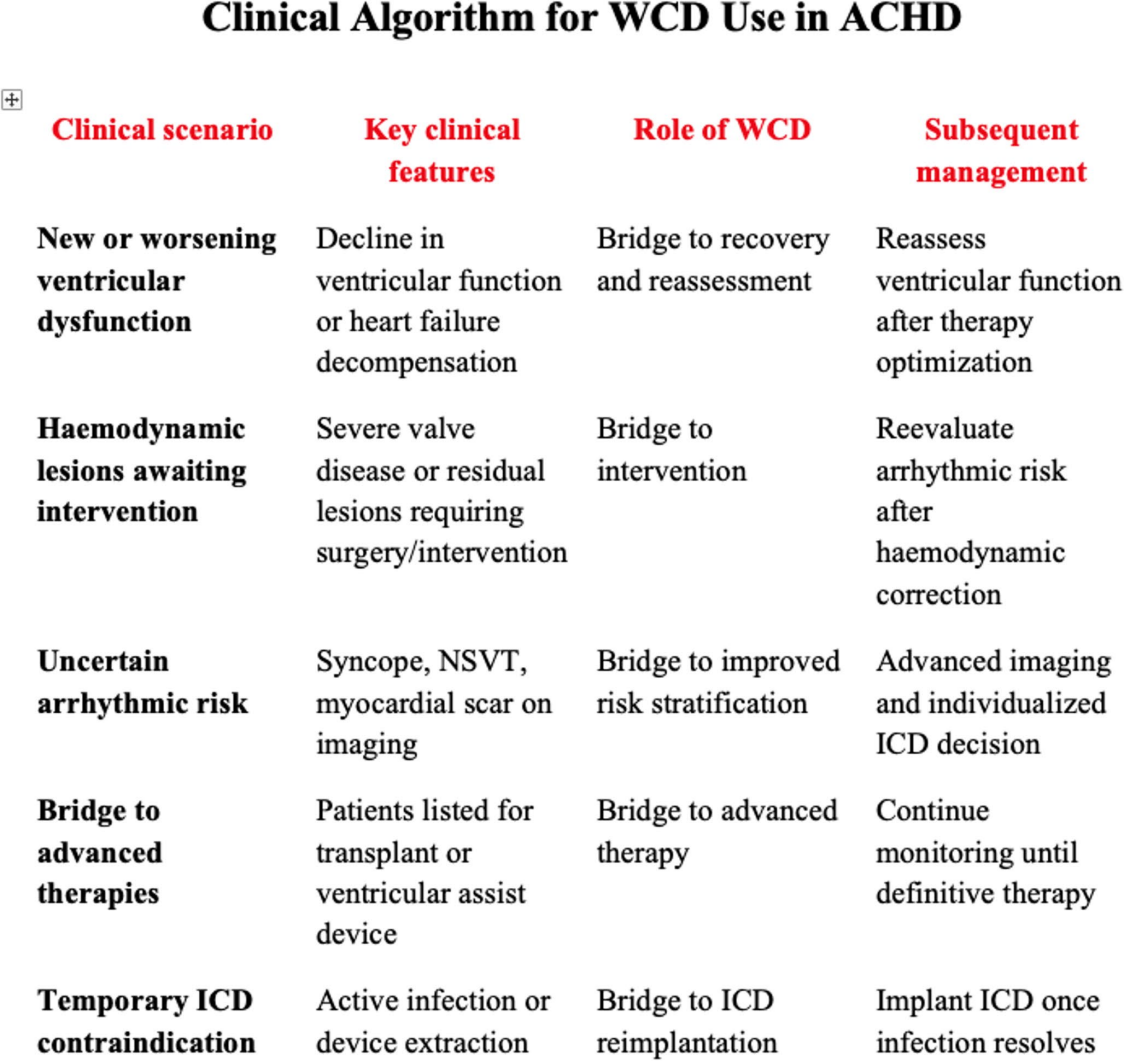
Clinical algorithm for WCD use in ACHD

In this context, the WCD should be considered a temporary and flexible tool that enables dynamic risk reassessment and supports individualized clinical decision-making (Fig. 4). Further multicentre studies and dedicated registries are needed to better define its role in ACHD management.

## Data Availability

No datasets were generated or analysed during the current study.

## Declarations

### Web-site

www.berardosarubbi.it.
